# Promoting Sustained Real-Life Benefits of Virtual Reality–Based Interventions in People With Mental Health and Substance Use Disorders: Qualitative Study

**DOI:** 10.2196/57643

**Published:** 2025-08-20

**Authors:** Jan Aasen, Fredrik Nilsson, Torgeir Sørensen, Marja Leonhardt

**Affiliations:** 1 Research Centre for Substance Use Disorders and Mental Health Disorders (ROP-Forsk), Innlandet Hospital Trust, Erik Werenskiolds veg 3, Hamar, Norway, 47 99428096; 2 Centre for Diaconia and Professional Practice, VID Specialized University, Oslo, Norway; 3 RIO - Rusmisbrukerenes interesseorganisasjon, Oslo, Norway; 4 Research Centre for Existential Health, Innlandet Hospital Trust, Ottestad, Norway; 5 Section for Clinical Addiction Research, Oslo University Hospital, Oslo, Norway

**Keywords:** mental health and substance use disorders, virtual reality–based interventions, immersive learning, cognitive load, social skills acquisition, template analysis

## Abstract

**Background:**

Concurrent mental health and substance use disorders (MHD/SUD) are one of the most prominent public health problems as of today, and the worldwide prevalence of MHD/SUD is currently increasing. Modern virtual reality technology may provide easy, unlimited, and safe access to social experiences and interactions that hold the potential to promote individuals’ new learning for the benefit of their social participation and recovery. However, the clinical adoption of virtual reality–based interventions (VRIs) is still in its infancy. Human limitations in skills transfer from virtual to actual reality are a major challenge in designing efficient VRIs. Key working mechanisms of the interactive, digital social environments in virtual realities have yet to be identified. There is a lack of knowledge on how immersive learning experiences may be designed and structured to promote sustained real-life benefits for people with mental health and substance use disorders.

**Objective:**

The main aims of this paper were to explain the factors affecting the outcomes of learning in multisensory virtual reality environments and to examine how they affect our particular target group. The overall purpose of this study was to understand how learning experiences in VRIs may be designed and orchestrated to promote sustained real-life benefits of VRIs in people with MHD/SUD.

**Methods:**

Eight individual in-depth interviews with adults in recovery from mental health and substance use disorders were conducted in a medium-sized municipality in eastern Norway in fall 2022. The interviews were analyzed using template analysis, a form of codebook thematic analysis, in a process involving peer researcher collaboration.

**Results:**

This study suggests that the human capacity to achieve sustained learning outcomes from multisensory immersive learning experiences was limited in general. This study also indicates that people with mental health and substance use disorders struggle with attention deficit, concentration, and memory to an extent that it affects their daily functioning.

**Conclusions:**

Altogether, the theoretical framework and empirical findings provide added information on how we may develop learning experience designs in VRIs that accommodate human perceptual processes. VRI scenarios that may be repeated and structured according to individual learning prerequisites may enable the restructuring of maladaptive social schema. This may possibly promote the storage of new, repaired schemas in the user’s long-term memory. It is therefore suggested that short, focused VRI scenarios, orchestrated in a sequenced and deliberately structured learning workflow, may promote sustained real-life benefits from VRIs in people with mental health and substance use disorders.

## Introduction

### Overview

Concurrent mental health and substance use disorder (MHD/SUD) is one of the most prominent public health problems as of today. The worldwide prevalence is currently increasing [1]. With the increasing prevalence of MHD/SUD globally, there is a need for developing more comprehensive, integrated, and evidence-based responses for harm reduction and support to recovery among people with MHD/SUD [1,2]. Simultaneously, the increasing demand for health and welfare services is a major concern for our future health and welfare services. Staff shortages are one of the most important factors threatening capacity going forward [3]. This leads to a need for understanding how the use of digital technologies, such as virtual reality–based interventions (VRIs), may be optimized in novel MHD/SUD treatment [1,2,4].

Focusing on social and functional recovery and supporting people in the process of becoming active and participating citizens are key parameters within the recovery process for people with MHD/SUD [5,6]. Modern virtual reality (VR) technology may provide easy and safe access to social experiences and interactions that hold the potential to promote individuals’ learning for the benefit of their social participation and recovery [7-10]. Summarized research shows that VRIs have few challenges in recreating sufficient realism in the simulations to provide emotional responses and sufficient learning outcomes in experimental and clinical settings [9,11,12]. Current research shows that VRIs may be equally or more efficient than conventional treatment. The highest evidence and application maturity for VRIs are for treating PTSDs and anxiety disorders [13,14]. Once developed, VRIs can be distributed to an infinite number of hardware devices without any significant increase in costs [13]. However, the clinical adoption of VRIs is still in its infancy [13]. VRIs hold the potential to strengthen treatment capacity, treatment availability, and treatment quality in future mental health care services [7].

Lack of personalization of VR apps to patient needs is regarded as a major impediment to clinical adoption [15]. A limited amount of research has been conducted to date on the efficacy of VR-based cue exposure approaches for addiction, which aim to extinguish craving and prevent relapses. Small trials on subjective and physiological outcomes have emerged for nicotine and alcohol use disorder and pathological gambling [13,16]. Many of these studies, however, lack sufficient methodological rigor, and further large-scale randomized controlled trials are missing [13].

The literature reflects a wide variation of VR environments and software apps, in which all of them are regarded as VR, but they evidently provide different user experiences [17,18]. There is also a wide variation in VRI duration, dosage, and repetition, with little knowledge on optimizing learning workflow to promote efficient and sustained learning uptake [19]. There is also a lack of theoretical conceptualization and understanding of the characteristics of effective VRIs [7,11,13,20]. There is a need for a better understanding of factors that may affect positive VRI outcomes [16].

The key working mechanisms in virtual realities in terms of clinical outcomes and behavioral change have yet to be identified [8,21,22]. A main challenge when developing efficient VRIs is human limitations of transferring acquired skills from digital to real environments and their long-term maintenance [8,9,12,23-26]. A nearly 41% increase in frontal theta power was observed when testing cognitive load using an electroencephalogram with a fully immersive head-mounted VR display, compared with a 2D semi-immersive tablet display [27]. This points to the focus on understanding the impact of immersive VR experiences on the human perceptual processes [28]. Challenges associated with human factors in immersive learning in general include general health, physiology, adverse effects, cognitive load, and compatibility [29].

However, all learners are not equally capable of effective and efficient learning [30]. The characteristics of a population group targeted by VRIs are important determinants. These influence the impact that digital interventions may have on their functional trajectories [31]. It is also vital to understand the target group’s perception of the use of digital interventions, as these are key uptake factors for all types of digital interventions as well [31].

Little research has been conducted on uptake factors for immersive learning or the feasibility of VRIs among people with MHD and SUD [20]. Studying key features, VRI uptake factors, and learning prerequisites among people with MHD/SUD is thus essential for creating VRIs that provide effective and persistent skills transfer from VR to real life.

### Aim of the study

There is a lack of explanations of the factors affecting the outcomes of learning in multisensory VR environments. Explaining these factors and examining how they affect our particular target group was the main aim of this paper.

This main aim led to 2 subaims for this study. The first aim was to obtain a better understanding of human perceptual processes in fully immersive VR in general. The second aim was to examine how these perceptual processes were affected among people with MHD/SUD.

The overall purpose of this study was to understand how learning experiences in VRIs may be designed and orchestrated to promote sustained real-life benefits of VRIs in people with MHD/SUD.

### Theoretical Framework

Learning experience design involves creating learning activities that induce an active process of constructing and retaining knowledge. This also includes orchestrating learning activities in a sequenced or otherwise carefully and deliberately structured learning workflow [30]. In immersive learning, the term “immersion” describes how technological affordances facilitate learning by inducing a sense of presence or copresence in digital environments. “Learning” refers to the response to immersive experiences in human perceptual and motor systems [28]. This indicates that a theoretical framework for the learning experience design in VRIs may be derived from basic principles of human learning. Within adult learning, this is referred to as didactics. The didactical aspect of social skills training in VRIs is important to ensure that the participants achieve the learning goals related to the determined learning objectives (knowledge, skills, attitudes, behavior) from the VRI scenarios. Didactics is an umbrella term for the theoretical and practical application of learning in a wider sense than teaching. The didactical foundation of adult simulation training is most commonly based on Kolb’s experiential learning theory [32,33]. Experiential learning is based on five crucial assumptions for adult learning: (1) adult learners are self-directed and self-regulated, (2) adult learners are intrinsically motivated to learn, (3) adult learners have previous knowledge and experience that form mental models which guide their behavior through this experience, (4) adults use analogical reasoning in learning and practice, and (5) one cannot force learning upon adults [33]. Adult learners must be explained the relevance the learning has to their work and guided in the understanding of how, when, and why to learn [33].

In VRIs, this learning process stems from constant multisensory inputs that depend on the individual’s perceptual capacity to respond to multiple stimuli simultaneously. This requires executive shifts in focused attention according to the demands of the particular VRI scenario. This relies on human perceptual processes. In VRIs, learners make sense of information by organizing sensory input into understandable forms. They built meaningful mental pictures from the information given in the VR setting. This is known as constructivist learning [34]. According to the constructivist learning theory [34], learning occurs when learners are able to build referential connections between corresponding aspects of the visual and verbal representations of the learning object. Constructivist learning is fostered when the learner is able to hold a visual representation in visual working memory and a corresponding verbal representation in verbal working memory at the same time [34]. This also implies that the cognitive load on the working memory acts as a major impediment to social skills learning in multisensory learning environments, such as the VR scenarios in VRIs [34].

Attention and short-term memory processing are thus abilities for constructing sustainable learning outcomes from multisensory learning experiences in VRIs. This is a key distinction as the learning conveyance determines the learning uptake, whereas the retaining process determines the durability of the learning uptake.

**Figure 1.**
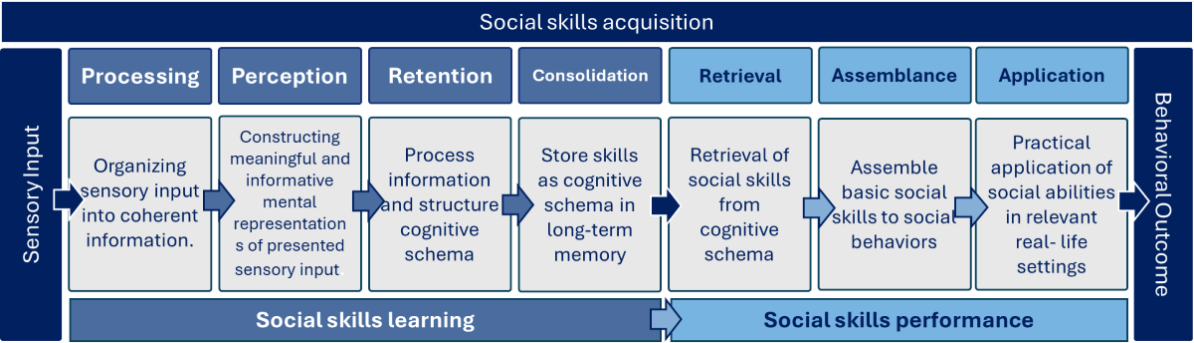
The process from sensory perception in virtual reality to practical application of new behavioral repertoires in real-world situations. Figure by authors, based on the description of social learning and constructivist learning in multimedia environments by Mayer et al [34] and Kopelowicz et al [37].

This is important for the ecological validity of the VRI. The ecological validity of VRIs refers to the extent to which VRI users improve functioning, daily life skills, compensatory strategies, social participation, and psychological well-being in real-life settings and situations [8]. The process from sensory perception to using new behavioral repertoires in real-world situations in social learning is referred to as social skills acquisition [35]. The concept of acquisition refers to how persons acquire abilities and construct knowledge through mental information [35]. This refers to how cognitive structures develop and change, and how repertoires of new behaviors are acquired, used as practical intelligence, and used in capabilities to behavioral adaptation in real-life settings [34,35]. Learning deals with how people construct knowledge through mental information and how cognitive structures develop and change. Social performance is the process of retrieving social skills from the cognitive schemas in long-term memory and applying these skills to appropriate behaviors in relevant real-life settings [34,36]. The acquisition process is illustrated in Figure 1.

**Figure 2.**
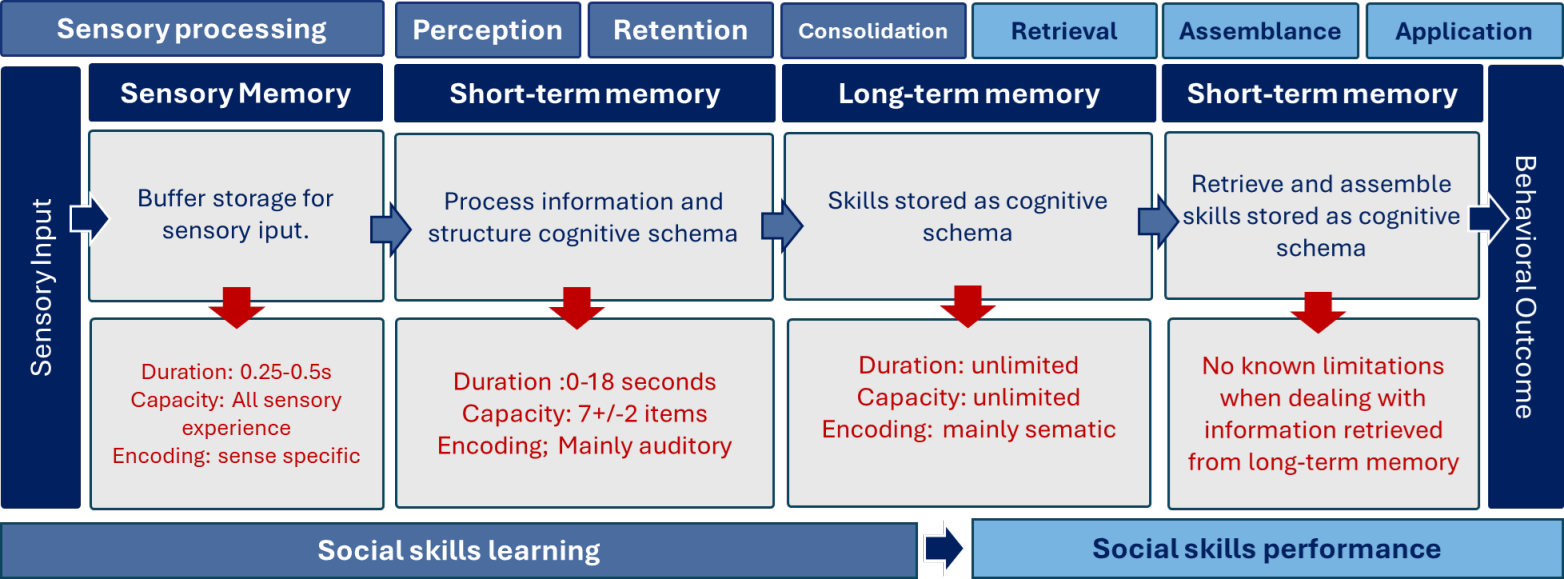
Cognitive limitations in the process from sensory perception in virtual reality to using new behavioral repertoires in real-world situations. Figure by authors, based on the description of social learning and constructivist learning in multimedia environments by Mayer et al [34], Kopelowicz et al [37], and Atkinson and Shiffrin [40].

The link between high scenario authenticity, real-life fidelity, and ecological validity has been a dominating assumption in the field of simulation-based learning [38]. Learning tasks are mainly designed on the basis of real-life problem-solving with complex element interactivity, under the assumption that this leads to effective task performance in real-life settings [39]. According to cognitive load theory, this is contradictory to the functionality of human cognition [38]. Cognitive overload in learning leads to a collapse in memory processing and thus also in skills acquisition. Cognitive load theory is based on the assumption that the human cognitive system has a limited working memory [38]. This assumption is derived from Atkinson and Shiffrin’s [40] classical work on multimodal memory. They explain human memory as a multimodal system consisting of sensory memory, a transient short-term memory buffer, often referred to as working memory, and a long-term memory store [40,41]. The working memory enables transient evaluation, manipulation, and synthesis of newly acquired information. The working memory operates within the short-term memory and interacts with attention and executive function [41]. According to Atkinson and Shiffrin’s [40] theory on multimodal memory, human working memory is unable to hold more than 5 to 9 information elements and can actively process no more than 2 to 4 elements at the same time. Additionally, all unprocessed information in the short-term memory is lost in about 20 seconds when dealing with novel memory obtained by information processing. Input that exceeds the sensory buffer capacity will be overwritten by new sensory inputs and forgotten before this sensory information is passed on for further processing [38]. In contrast to processing sensory input, short-term memory has no known limitations when dealing with information retrieved from long-term memory. Only skills stored as cognitive schemas are available and sustainable in long-term memory [38]. The cognitive limitations in the acquisition process are illustrated in Figure 2.

The long-term memory holds cognitive schemas that vary in their degree of complexity and automation. These schemas can then be treated as a single element in short-term memory. This greatly decreases the cognitive load associated with the performance of later tasks. Practicing skills already stored in long-term memory allows for a far higher task load than novel skills learning [38]. The cognitive load within the skills acquisition process is divided into 3 distinct cognitive load modalities: extraneous, intrinsic, and germane load [38]. Extraneous load is load generated by external factors that interfere with cognitive processing, negatively impacting the learner’s capacity to buffer and process the relevant sensory inputs. Intrinsic load is load directly related to the material or task given in the learning situation and is often defined by the number of individual elements and interactivity between them. Germane load is the load on short-term memory capacity generated by accessing mental schemas and making connections necessary to move the material to long-term memory [38]. As the total cognitive load accumulates throughout the acquisition process, reducing the extraneous load will free up cognitive capacity for further perceptual information processing [38,39]. Unnecessary information in the VRI scenarios may thus hamper the end users’ skills acquisition.

## Methods

### Context and Design

For this qualitative exploration, we used data from 8 in-depth interviews with people in recovery from MHD/SUD. The interviews were conducted in a medium-sized municipality in Eastern Norway during the fall of 2022.

### Recruitment

Recruitment for this study was conducted at a nongovernmental support center for people in MHD/SUD recovery. The first author presented the research project to the whole group of service users present and invited them to participate in the study. Those who wished to participate then scheduled interviews with the staff of the center they attended. The inclusion criteria were as follows: (1) aged 18 years and older, (2) self-reported MHD/SUD with experience of social marginalization, and (3) the capacity to understand the study information and informed consent. This sampling strategy is a type of convenience sampling called volunteer sampling, where the decision to participate is strongly dependent on respondents themselves due to the nonindividualized nature of the invitations [42]. Convenience sampling is a sensitive recruiting strategy as we met all the participants in their own settings and caused little disturbance.

### Participants

Eight persons with self-reported MHD/SUD, 3 females and 5 males, were recruited. The average age of the participants was 48 years, ranging from 42 to 61 years. One participant was an Eastern European immigrant, and one participant was not a native Norwegian but had grown up with Norwegian foster parents. The other participants were native Norwegians. Seven participants started substance use in early adolescence, with an average age of onset of 12 years. Six participants were in opiate agonist treatment at the time of the interviews. One participant reported polysubstance use without opiate dependency, and one participant reported opiate dependency only. All participants reported MHD experience, including anxiety, depression, attention deficit/hyperactivity disorder, posttraumatic stress disorder (PTSD), and personality disorders. All participants reported prior experienced social marginalization due to MHD/SUD. The participant demographics are presented in Table 1.

**Table 1.** Participant demographics.

Participant	Sex	Age (years)	SUD^a^ onset (years)	Self-reported MHD^b^
1	Male	51	44	PTSD^c^, ADHD^d^, and anxiety
2	Male	42	11	PTSD, ADHD, anxiety, and psychosis
3	Male	50	11	Anxiety
4	Male	44	12	Complex PTSD, ADHD, personality disorder, depression, and psychosis
5	Female	51	10	Anxiety
6	Female	43	13	PTSD and anxiety
7	Female	61	10	Anxiety
8	Male	45	14	PTSD/ ADHD/Anxiety

a SUD: substance use disorder.

b MHD: mental health disorder.

c PTSD: posttraumatic stress disorder.

d ADHD: attention deficit hyperactivity disorder.

### Data Collection

The first author conducted the first 3 interviews independently, while the other 5 were comoderated by the first and second authors. The interviews lasted from 24 to 90 minutes and followed a semistructured interview guide. The interview guide consisted of open-ended questions on how the participants perceived their opportunities and prerequisites for participating in work, education, and cultural life in mainstream society. In order to ensure a comfortable and safe setting for the participants, we conducted our interviews at the support center where the participants were recruited. All the interviews were audiotaped and transcribed verbatim. The second author is a peer support worker who has lived experience of MHD and SUD. He participated in developing the interview guide, conducting the interviews, and collaborating in the analysis.

### Analysis

Data were analyzed using template analysis with a deductive approach [43]. Template analysis is a variant of codebook thematic analysis [44,45]. Template analysis is theory-led and based on a set of a priori themes organized in a predetermined template. Key uptake factors for immersive learning were derived from the present theoretical construct and used as a priori themes. A priori themes are often based on theoretical ideas that have guided a particular study, as in this study [43].

The present theoretical framework implies that perceptual capacity, working memory, and executive functioning are key prerequisites for learning uptake from multisensory learning experiences in VRIs. Additionally, prior social modeling is a key factor for prior schema development. Hence, cognitive schemas act as key preconditions for social skills learning, as these schemas determine whether the users need to learn novel skills or practice skills already stored in the long-term memory. The a priori themes were developed from these theoretical principles. The thematic template is presented in Table 2.

In the first step of the template analysis, the first author familiarized himself with the data by exploring the verbatim transcriptions of the interviews and by reading the transcriptions afterwards. In the second step, the first and second authors generated initial codes from empirical statements within and across the transcripts.

The first author then presented the empirical statements and initial coding to the other authors in step 3. In step 4, the meaning and relevance of the empirical statements were discussed at both the semantic and latent levels with all authors. Step 5 consisted of the first author interpreting the data using the template. The results were then discussed with the second and fourth authors in step 6. Additionally, the empirical findings were presented, discussed with, and verified by the interview participants. The first author wrote up the analysis in step 7. The third and fourth authors edited the analytic description, and the first author drafted the final report of the results.

In this analysis process, the first author brought philosophical meta-theoretical assumptions, prior professional experience as a specialized psychiatric nurse, and theoretical knowledge to the analysis. The second author brought an additional layer of analytical perspective with lived experience, forming his preunderstandings in the shaping of the codes and revision of the themes. The themes are validated by the respondents, all themes are connected to quotes, and all participants are represented in the results. The themes, theme relations, theme hierarchy, and result descriptions are discussed and validated by the research team and the study participants.

**Table 2.** A priori themes and a priori subthemes.

	A priori themes	A priori subthemes
1	Digital uptake factors	Digital literacy Digital intervention receptiveness VRI^a^ expectations
2	Immersive learning capabilities	Executive functioning Memory processing Cognitive capability
3	Social skills learning preconditions	Prior social skills modeling

a VRI: virtual reality–based intervention.

### Ethical Considerations

People with MHD/SUD are a highly vulnerable and particularly hard-to-reach population, which requires particularly considerate and gentle collaboration with our participants during recruitment and interviews. Within this target group, many are struggling with social anxiety and low trust in strangers. All recruitment and data collection in this study were conducted in line with the Norwegian guidelines for medical and health research. This study was approved, pursuant to the Norwegian Health Research Act, §10, by the South-Eastern Norway Regional Committee for Medical and Health Research Ethics (ref. 421376), and the Data Protection Officer of Innlandet Hospital Trust (ref. 18197741). All participants signed an informed consent that described the purpose of the project, personal data processing requirements, and data protection plan. The participants were allowed to withdraw from the study at any time. The researchers discussed potential vulnerability within the context of the research, potential consequences of their research participation, and potential positive impact of the research with each participant before signing the consent to participate. The participants were also informed about their right to withdraw their data from the research. The participants were not compensated for their study participation. The research data in their final version, after coding and deidentification, is stored in the form of text files (doc./docx.) and audio files (MP3). During the data collection, data processing, and analysis phases of the project, research data are stored in the researchers’ personal, password-protected areas at the Innland Hospital Trusts’ secured research server. At the end of the project period, the research data will be archived at Services for Sensitive Data (TSD) at the University of Oslo, in accordance with current agreements with Innlandet Hospital Trust and VID Specialized University. When archiving data, each dataset will have an identifier (personal identification).

## Results

### Overview

Three main themes were derived from the analysis based on the thematic template: (1) VRI receptiveness, (2) deficient immersive learning abilities, and (3) disadvantageous social learning prerequisites. The overall theme structure with a priori themes, final themes, and the associated subthemes is presented in the final thematic map in Table 3.

In the following, the main themes and the codes will be explained and related to empirical statements from the participants. These are presented in italics to show how the content appeared in the interviews. The statements have been translated from Norwegian with the original meaning preserved as far as possible.

**Table 3.** Final main themes and the associated subthemes.

	Final main themes	Final subthemes
1	Digital uptake factors	Digital literacy Digital intervention receptiveness VRI^a^ expectations
2	Immersive learning capabilities	Executive functioning Memory processing Cognitive capability
3	Social skills learning preconditions	Prior social skills modeling

a VRI: virtual reality–based intervention.

### Theme 1: VRI Receptiveness

This theme refers to the participants’ digital intervention uptake factors. This includes experience with digital technologies in general and VRT in particular. In general, the participants reported various levels of digital literacy. Some participants reported little experience with using computers and expressed a perceived lack of digital literacy in their daily lives.

These participants reported difficulty in using banking apps and web portals for public services. They also lacked access to digital technologies:

*I haven’t learned much computer stuff. I should though, otherwise you’ll get stuck in today’s society.*

[P6]

Other participants used computers on a daily basis in work practice and reported average user competence. Others reported extensive experience with gaming and serious interest in digital technology:

*I spend much of my days on Netflix and gaming […].*

[P8]

With regard to VRT in particular, the participants reported having heard of VR and having understandings and opinions about VR and VRTs. They all had seen head-mounted VR displays, and some had experience of using recreational VR apps.

*I have those cheap VR goggles at home, which I can put my phone inside, then I sit at the bottom of the sea watching sharks and so on.*

[P3]

This theme also accommodates the participants’ attitudes towards meeting technology-based interventions instead of those delivered by humans in MHD and SUD treatment. The participants with little digital experience reported interest in the possibility of new kinds of digital interventions to promote their recovery.

Some participants described how their social anxiety was triggered by human presence, regardless of the situation and their relationship with the person present. One participant explained how she felt that digital interventions could create a safe social learning space to benefit people who struggle with social anxiety:

*Well, I think VR goggles would be really effective for me. So, I can sit at home and practice social skills so that I don’t have to think about what I’m doing and what I’m saying and how I’m standing, because then I can focus on the things I want to focus on and not all the nonsense that I constantly get hung up about. So actually, I think VR would be very effective for me.*

[P6]

Overall, the participants expressed positive attitudes towards VRI and were receptive to using VRIs as a tool for social skills learning.

### Theme 2: Deficient Immersive Learning Abilities

This theme is related to the participants’ abilities to acquire social skills through immersive learning experiences. The participants described a wide range of social and functional impairments in their daily lives. Their difficulties varied, but they all reported problems with concentration and short-term memory to an extent that impaired their everyday functioning.

Remembering appointments and messages was difficult for all the participants. They related many of their daily challenges to experiences they reported about difficulty in regulating attention, remembering things, or concentrating on tasks. They reported more profound impairments, such as the inability to recognize people and retain knowledge. The participants narrated their everyday struggles in different ways:

*I cannot concentrate... I can’t even read books, and I love to read.*

[P1]

*Well, I forget things, like if I’m just going to get something from the kitchen, I have to go back because I forget what I was supposed to be doing.*

[P3]

These quotations illustrate the participants’ attention deficits and short-term memory. Several of them reported better functioning prior to starting serious substance use and elaborated on how their mental capacity had deteriorated due to excessive polysubstance use and possible hypoxic brain damage from multiple overdoses:

*[…] I reckon I’ve messed up quite a few things in my head because of my substance use, so I’m very forgetful.*

[P2]

*I’ve had 36 overdoses when I was given an antidote, so I should have been dead a long time ago.*

[P8]

The participants claimed some improvement in their cognitive deficits after abstaining completely from substance use for an extended period. However, none of them had experienced a full recovery of their cognitive abilities. Several of them talked about how they did not understand the impact of sobriety until they became entirely sober. They described how abstaining totally from substance use had been important for their functioning and well-being.

They also reported that being clean was vital for benefiting from learning-based interventions. One of the participants expressed it in this way:

*You have to give up powder and cut out tablets, the most important thing is you have to abstain from substance use enough to get to a point where you’re able to think.*

[P7]

The participants also talked about how opioid agonist therapy affected them in their everyday functioning. One participant explained his situation as follows:

*I need to get off that methadone and all that stuff because it limits me so much in everything — I sweat and get tired and get totally worn out — just from washing up my dishes.*

[P3]

### Theme 3: Disadvantageous Learning Prerequisites

This theme describes the life experiences that have formed the participants’ cognitive schemas for social skills and that determine their social learning preconditions. Some of the participants described adverse childhood experiences, such as neglect, domestic violence, substance use, or lack of empathic responses from their caregivers. Others described warm relations with their parents and siblings, but harsh social treatment with bullying and exclusion among their peers and in school. One of the participants explained:

*We lived quite a sheltered life when I was little…until I started elementary school. There the whole school bullied me, and when I got home my father gave me a beating […] it was a lot like that.*

[P3]

Additionally, the participants described many adverse experiences during their lives in substance use communities. Due to substance use onset early in their preadolescence, they were associated with their local substance use communities from a very young age. They described these scenes as harsh environments that exposed them to deviant behavior, such as violence, exploitation, and intimidation:

*It’s a kind of environment, on the streets, that comes with a lot of stuff…*

[P8]

Several of the participants also stated that when they came off substances, they found themselves at the same socioemotional stage as when they started using them. They explained that they were still struggling with immature socioemotional functioning and described a perceived lack of adult behavioral repertoires. One participant put it this way:

*[…] now I’ve just woken up from twenty-four years of substance use […] and I notice that I have the same mental problems I had when I was fourteen.*

[P7]

The participants spoke about adverse school experiences. Several described learning difficulties and little help from teachers. They also talked of social exclusion and bullying in elementary school. They often skipped classes, and most of them were early dropouts. Overall, the participants expressed that they were missing much of the emotional and social skills they believed were required for appropriate adult functioning.

The participants also perceived their current social abilities as unfit for participation in mainstream society among people they considered normal. One of them summed it up in this way:

*[….] you kind of have to learn everything all over again, learn to be human and relearn how to live.*

[P6]

## Discussion

### Principal Findings

This study sought to provide a better understanding of the human perceptual processes in immersive VR and how these key VRI uptake factors were affected among people with MHD/SUD. This study suggests that the capabilities for social skills acquisition in VRIs are limited in people in general. The empirical findings in this study suggest that the immersive learning capabilities of people with MHD and SUD are inferior to those of the population in general. This study also showed disparities in digital literacy among the participants, but they were all positive towards using VRIs to promote their social participation and functional recovery.

The participants reported various levels of experience with digital technologies, but they all knew what VR was. They were receptive to meeting digital interventions instead of human-delivered measures in MHD and SUD treatment. The participants also suggested that digital interventions could alleviate social anxiety in social skills training, as such interventions enable social skills learning without the presence of humans, which triggers social anxiety. Overall, this study suggests that VRIs may be acceptable and feasible to use with people with MHD/SUD. This is in line with a systematic review on the feasibility of digital interventions among MHD and SUD populations, which shows that VRIs are feasible, but not consistently effective [20]. A study on the feasibility of VRIs for people who experience psychosis shows that they are feasible and are accepted by this patient group [9].

A key finding in this study is how the participants expressed difficulty with attention, concentration, and memory to an extent that it affected their daily functioning. Previous research shows that MHD and SUD often consist of various combinations of mental health disorders, neurodevelopmental disorders, PTSD, and long-term substance use complications. This can lead to substantial cognitive impairment [46-48]. Neuropsychological research from the last 2 decades has consistently demonstrated that impairments in cognitive functioning are frequent among people with substance use disorders [49]. It has also been found that a majority of people with MHD and SUD struggle with impaired learning prerequisites in various forms and severities [47].

Deficits in executive functioning, decision-making, and goal-directed behaviors are the most prominent problem areas observed in people with problematic substance use [49-51]. This includes difficulty with attention, planning, memory, problem-solving, and self-regulation. All these factors affect daily functioning, including the capacity to understand concepts and skills learned in an intervention and apply them to daily lives [50]. Tasks that measure working memory have revealed cognitive deficits in individuals with substance use disorders [52]. Working memory refers to the capacity to monitor and alter information held in mind temporarily, inhibition involves overriding an unwanted distraction to maintain task focus, and shifting pertains to flexibly switching attention between tasks or mental sets [53].

Deficits in short-term memory were also a prevalent topic among the participants. Short-term memory processing relies on attention and is crucial for constructing and reconstructing cognitive schemas that can be stored in long-term memory [38]. Attention is key to awareness and focusing on a stimulus, or a set of sensory stimuli. Attention is also key to the perception, selection, and filtering of sensory input [41]. Sustained attention is the capacity to maintain attention over an extended period, whereas selective, or focused, attention is the ability to preferentially attend to a subset of stimuli [41]. VRIs with head-mounted displays isolate the user from external distractions and are thus well suited for catching the VRI user’s attention. However, the learning outcomes in VRIs also rely on divided attention.

The constructive learning process in VRIs stems from constant multisensory inputs that depend on the individual’s capacity to respond to multiple stimuli simultaneously. This requires executive shifts in focused attention according to the demands of the particular VRI scenario [34,41]. Attention and short-term memory processing are thus paramount abilities for constructing sustainable learning outcomes from multisensory learning experiences in VRIs. Cognitive load theory indicates that in VRIs, sensory input that is not directly involved in the learning conveyed may worsen rather than promote the desired learning outcome [39]. Hence, limiting cognitive tasks in VRI scenarios for social skills learning is likely to promote the transfer of skills from digital to real environments. VRI scenarios that only expose the user to sensory input necessary to convey particular learning goals are therefore likely to promote efficient learning uptake.

Another key finding in this study is the participants’ descriptions of their childhood and adolescence. The participants reported multiple experiences of adverse life events from early childhood, as well as exposure as adolescents to deviant social behavior in substance use environments. This explains some of the problems the participants described with social participation in mainstream society. Adverse childhood experiences are one of the most robust predictors of substance use and are commonly found among people with MHD/SUD [54,55].

The participants’ reports of violence and intimidation in substance use environments are also in line with previous research [56,57]. Adverse childhood experiences and other deviant social experiences heavily affect the development of individuals’ cognitive schemas. This is commonly found in people with MHD and SUD [58]. Adverse childhood experiences include a set of highly correlated traumatic and negative events that include child maltreatment (eg, sexual, physical, and verbal abuse) and household dysfunction (eg, parental divorce, familial substance use, mental illness, and incarceration) [54]. The cognitive schemas for interpersonal relationships that specify what to expect from others and how to treat them are formed during preadolescence, in particular. Young people living in stressful or harmful environments can form atypical schemas that enable them to survive adolescence but lead to serious problems when they are applied to adult relationships later in life [58].

Preadolescents exposed to stressful or otherwise difficult interpersonal environments where their emotional needs are not met develop dysfunctional beliefs about themselves and the world. Over time, this can lead to cognitive and emotional impairments that directly affect learning abilities through neurocognitive pathways [54]. When the onset of substance use and mental illness occurs before adult social skills are learned through natural processes, such as those the participants described, individuals will often form dysfunctional and maladaptive cognitive schemas for the requisite skills for independent functioning and personal coping. Maladaptive schemas cannot be altered or adapted to new situations or new social contexts in the same way as functional schemas. With accumulated exposure to emotional stress and a lack of empathic responses throughout the adolescent’s development, these schemas become more complex, dense, and rigid. Hence, they also become increasingly difficult to change [58,59]. This hampers the capability of developing social abilities and adapting them to new social circumstances, compared with people with functional and adaptive cognitive schemas.

Hence, people with MHD and SUD often have serious difficulty in adopting appropriate social behavior in society at large. This is a key finding in regard to VR-learning experience design, as people’s maladaptive cognitive schemas require restructuring or overwriting in order to acquire and retain social skills [58]. Overwriting or restructuring old cognitive schemas or constructing new ones requires repeated practice and overlearning to ensure the assimilation and retention of social skills. Such learning is facilitated when errors are minimized and correct responses are strengthened with abundant positive reinforcement [37].

### Strengths and Limitations

This study comes with some limitations. A major limitation of this study is that our limited sampling did not encompass the full range of disparities in our multifaceted target group. This study was aimed at a particularly vulnerable population, which is traditionally hard to reach. The sample size (n=8) is small, but it is within Braun and Clarke’s [60] recommendation of 6‐10 participants for small projects. The empirical findings in this study are in line with a number of previous studies and widely supported in the field of mental health and substance use care [49,61,62]. It is also found that cognitive impairment affects daily and social functioning across the entire range of psychiatric diagnoses [41,47]. Altogether, this suggests the sample size was adequate to meet the research purpose of this study. Another limitation of this study is the demographic context.

This study was conducted in a Nordic welfare state. Thus, generalization to different demographic contexts may be limited since social participation in society will always depend on civil rights and social opportunities, as well as the individual’s social capabilities. The main strength of this study is the human-centered design process and the peer-researcher collaboration throughout the research process. The interviews with 2 interviewers, using a peer researcher as primary interviewer, also made a valuable contribution to the depth of our interviews and the richness of our data.

### Conclusions

Altogether, the theoretical framework and empirical findings provide new information on how we may develop VRIs that accommodate limitations in human perceptual processes. Regarding uptake factors for VRIs in people with MHD/SUD, this study showed wide disparities in digital literacy. However, the participants expressed receptiveness to VRIs, regardless of their digital literacy. The present empirical data and prior research show that this group has deficient immersive learning abilities and disadvantageous social learning prerequisites. Minimizing cognitive load in the VRI scenarios and orchestrating the VRI scenarios in a deliberate learning workflow may promote sustained real-life benefits from VRIs in people with mental health and substance use disorders.

### Practical Implications

The findings in this study suggest that people with MHD/SUD require precision learning, minimized cognitive load, and spaced repetition to achieve social skills that are constantly relevant for use in real-world situations. VRI learning experience designs may hence be aimed at errorless learning, structured in short scenarios that can be repeated until the desired learning outcome in each scenario is achieved by applying the principles of micro learning and spaced repetition to the learning experience design. Microlearning involves a careful task analysis of the required VRI learning content to identify specific learning units. When the required main learning units have been identified, the identified learning goals are broken down into short, focused units of learning content, called chunks or micro units [63]. These micro units constitute the learning goal of each scenario for learning social skills in the VRI. Short, focused VRI scenarios tailored to convey one micro unit of learning in a low-fidelity environment with decreased element interactivity in order to minimize cognitive task load may promote learning uptake [39,63].

Chunking VRI learning goals into microscenarios that can be repeated and structured according to individuals’ learning abilities and prerequisites may enable the restructuring of maladaptive social schemas and promote the storage of repaired and new schemas in the VRI users’ long-term memory. Ebbinghaus [64] showed that rehearsals should be distributed over time to become more efficient. Instead of repeating a task many times in the same session, it is more efficient to reduce the number of repetitions and spread them over several sessions [64]. When the users have achieved their required social skills, they will be able to benefit from practicing these skills in more complex VRI scenarios, aimed at applying adequate social skills to relevant social situations simulated in high-fidelity scenarios with high-reality proximity. This may enhance the learning sustainability, which in turn is vital for VRI’s learned skills to be available in real-life settings. This way of learning and practicing social skills may enable VRI users to further develop and adapt their new cognitive schemas based on real-world social experiences in the same manner as people with functional and adaptable cognitive schemas.

## Abbreviations

MHD: mental health disorder
PTSD: posttraumatic stress disorder
SUD: substance use disorder
VR: virtual reality
VRI: virtual reality–based intervention
