# Correction: Promoting Sustained Real-Life Benefits of Virtual Reality–Based Interventions in People With Mental Health and Substance Use Disorders: Qualitative Study

**DOI:** 10.2196/95127

**Published:** 2026-04-20

**Authors:** Jan Aasen, Fredrik Nilsson, Torgeir Sørensen, Marja Leonhardt

**Affiliations:** 1 Research Centre for Substance Use Disorders and Mental Health Disorders (ROP-Forsk), Innlandet Hospital Trust, Erik Werenskiolds veg 3, Hamar, Norway, 47 99428096; 2 Centre for Diaconia and Professional Practice, VID Specialized University, Oslo, Norway; 3 RIO - Rusmisbrukerenes interesseorganisasjon, Oslo, Norway; 4 Research Centre for Existential Health, Innlandet Hospital Trust, Ottestad, Norway; 5 Section for Clinical Addiction Research, Oslo University Hospital, Oslo, Norway

In “Promoting Sustained Real-Life Benefits of Virtual Reality–Based Interventions in People with Mental Health and Substance Use Disorders: Qualitative Study” [1], the authors noted one error.

Author JA’s affiliations have been revised from the following:

^1^Research Centre for Substance Use Disorders and Mental Health Disorders (ROP-Forsk), Innlandet Hospital Trust, Hamar, Norway

The author’s affiliations now read:

^1^Research Centre for Substance Use Disorders and Mental Health Disorders (ROP-Forsk), Innlandet Hospital Trust, Hamar, Norway

^2^Centre for Diaconia and Professional Practice, VID Specialized University, Oslo, Norway

The correction will appear in the online version of the paper on the JMIR Publications website, together with the publication of this correction notice. Because this was made after submission to PubMed, PubMed Central, and other full-text repositories, the corrected article has also been resubmitted to those repositories.
